# Diet culture mindset and meat restriction: A mixed methods mediation analysis

**DOI:** 10.1016/j.joclim.2025.100461

**Published:** 2025-06-10

**Authors:** Marina F. Jiao, Saadatu Abdul-Rahaman, Michelle Leonetti, Lizzy Pope, Kelsey Rose, Emily H. Belarmino

**Affiliations:** a Department of Nutrition and Food Sciences, University of Vermont, Burlington, VT, USA; b Food Systems Program, University of Vermont, Burlington, VT, USA; c Gund Institute for Environment, University of Vermont, Burlington, VT, USA

**Keywords:** Weight-bias, Plant-based diet, Vegetarian

## Abstract

**Introduction:**

Reducing meat consumption and eating a “plant-based diet” is recommended to improve health and environmental outcomes including reducing climate change. One understudied aspect of this dietary recommendation is how it may intersect with diet culture, which equates thinness to health. The purpose of this study is to explore the relationship between diet culture beliefs and meat limitation, and the potential mediating role of dietary motivation.

**Methods:**

This mixed methods study involved a national U.S. survey of 2,750 rural adults and interviews with 28 adults in Vermont, U.S.A. who limit meat intake. Survey respondents were asked about their dietary pattern, dietary motivations, and beliefs about “good” vs. “bad” foods and fatness. Responses were analyzed using mediation models. Interviews focused on perceptions of and experiences with plant-based eating. Transcripts were thematically coded and analyzed for diet culture beliefs.

**Results:**

Moralizing food and meat limitation were closely related. Health motivations fully mediated the relationship, while weight motivations partially mediated the relationship. Anti-fat beliefs were not related to meat limitation. Over half of interview participants imparted a moralization of “good” vs “bad” onto foods and nearly half discussed the healthfulness of plant-based diets and meat limitation.

**Conclusion:**

These results suggest that food moralization and meat limitation are related, and that individuals’ motivations to lose weight and be healthy may mediate the relationship. Although plant-based diets have potential health and environmental benefits, framing these dietary patterns around restriction may adversely affect eaters’ relationships with food. Further research with more diverse samples is needed.

## Highlights

• Limiting meat may support planetary health but could be linked to disordered eating.
• We examined meat limitation and diet culture beliefs among a US sample.
• Individuals with health and weight loss goals were more likely to limit meat intake.
• Moralizing foods was related to meat limitation.
• There is a need to consider diet culture risks when promoting plant-based diets.

## 1 Introduction

Food choices have long been socially moralized. Moralization guides eating behaviors through external pressure and the internalization of moralized ways of valuing food, which often leads to avoiding certain foods [1]. Moralization is central to “diet culture”, or the ubiquitous nature of dieting norms and expectations surrounding the idea that thinness is ideal [2]. Historically, the promotion of dieting has focused on caloric restriction and highlighting thin bodies. However, in recent years, the framing has shifted towards eating healthier and restricting certain food groups [2,3].

Meat consumption is increasingly the focus of morality debates [4] that center on health and environmental arguments [[5], [6], [7]]. Red meat in particular is a major contributor to key environmental burdens such as greenhouse gas emissions and land use, and a prominent risk factor for chronic disease and premature mortality. “Plant-based” eating patterns that limit meat are now widely recommended [8] and over a quarter of U.S. adults report limiting meat intake [9]. One reason suggested for the rise in meat limitation and the adoption of more restrictive plant-based diets (e.g., vegetarian and vegan) is their association with health-motivated dieting and thinness [10]. The aim of the present study is to explore the relationship between diet culture beliefs and meat limitation, and the potential mediating role of dietary motivation.

## 2 Methods

### 2.1 Survey research

An online survey related to meat limitation was developed and administered to a national sample of rural adults between July 2023 and June 2024. Individuals aged ≥18 years who had lived in the rural U.S. for ≥5 years were recruited through paid advertisements on Facebook and emails sent to members of a commercial email list purchased from Exact Data. Respondents were eligible to be entered into a raffle for a gift card. Data were retained from 2,727 individuals who completed at least 10 % of the survey and reported their dietary pattern. This study was approved by the Institutional Review Board at the University of Vermont (protocol ID#00002636).

Respondents were asked to provide their dietary pattern (omnivore, flexitarian, pescatarian, vegetarian, or vegan) and their motivation, with relevant response options being weight and health. Diet culture beliefs were assessed with two questions [11]: “I put a lot of effort into resisting bad foods” and “Fat people are unhealthy”. Respondents were asked to rank their agreement with the statements on a scale of 1–5. Responses to dietary pattern, diet culture, and demographic questions were converted to binary variables (Supplemental File 1, Table S1).

**Fig. 1:**
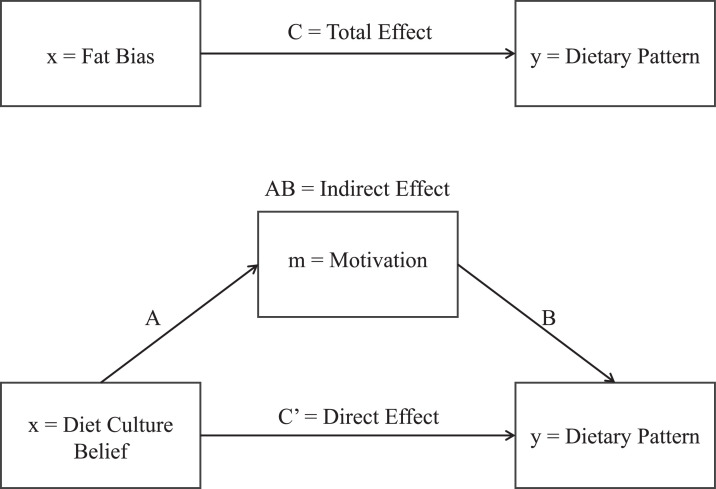
Relationships between selected diet culture beliefs and meat limitation, as mediated by health or weight motivations.

Data were analyzed in RStudio (Version 2024.4.0.735. Boston, MA: Posit team (2024)). The R medflex package for mediation analysis [12] was used to fit the binary data to a natural effects model and run multiple probit regression analyses using the counterfactual framework. The significance of natural effects was assessed using analysis of deviance (Supplemental File 1, Table S2). Fig. 1 presents the two models which examine agreement between each diet culture statement and meat limitation, as mediated by health or weight motivations. Relevant covariates (education, gender, and age) were identified by performing binary regressions with each individual demographic variable (Supplemental File 1, Table S3). For each model, only respondents that answered all questions relevant to that model were used resulting in 2,214 respondents for models involving “I put a lot of effort into resisting bad foods” and 2,203 for models involving “Fat people are unhealthy”.

### 2.2 Interviews

Twenty-eight rural adults from Vermont, U.S.A. who self-identified as healthy plant-based eaters were recruited and interviewed in 2022 to understand their food-related beliefs and experiences [13]. Participants completed a brief demographic and diet survey online before taking part in a phone or video conference interview using an interview guide based on the Theory of Planned Behavior [14] (Supplemental File 2). Participants were compensated with a gift card.

Interviews were audio recorded, transcribed, and checked for accuracy before being imported into NVivo (QSR International Pty Ltd. Version 20.1.6.1) for coding and analysis. For this analysis, interviews were open-coded by MJ for ideas and comments relevant to diet culture, such as weight status, restricting foods for health, and dieting for weight loss or body satisfaction [15]. Regular meetings were held to consider coding decisions. Code memos summarizing key themes were written by MJ and discussed with other team members. Qualitative themes were then integrated with quantitative results.

## 3 Results

Survey participant characteristics are presented in Table 1. Over one-third (37.4 %, *n* = 859) reported limiting meat intake. About half (51.5 %. *n* = 1183) said that their dietary pattern was motivated by health and nearly one in five (18.2 %, *n* = 418) said that they were motivated by weight. Two-fifths of participants (43.3 %, *n* = 996) agreed with the statement “I put a lot of effort into resisting bad foods” and over one-third (36.7 %, *n* = 843) agreed with the statement “Fat people are unhealthy”.

**Table 1:** Demographic and dietary characteristics.

Characteristic	All Participants (*n* = 2,299)	Non-meat limiters (*n* = 1,440)	Meat limiters (*n* = 859)	p-value
**Age**				
<43 years	947 (41.2)	518 (36.0)	429 (49.9)	<0.001
>43 years	1,352 (58.8)	922 (64.0)	430 (50.1)	
**Gender**				
Not a woman	851 (37.0)	585 (40.6)	266 (31.0)	<0.001
Woman	1,448 (63.0)	855 (59.3)	593 (69.0)	
**Education**				
Up to an associate's	1,030 (44.8)	721 (50.0)	309 (36.0)	<0.001
Bachelor's or more	1.269 (55.2)	719 (50.0)	550 (64.0)	
**Income**				
Less than $75 k	1,342 (58.4)	864 (60.0)	478 (55.6)	0.03
$75 k or more	928 (40.4)	556 (38.6)	372 (43.3)	
Did not disclose	29 (1.26)	20 (1.4)	9 (1.0)	
**Racial Identity**				
White	1,984 (86.3)	1,235 (85.8)	749 (87.2)	0.90
BIPOC	256 (11.1)	161 (11.2)	95 (11.1)	
Did not disclose	59 (2.6)	44 (3.0)	15 (1.7)	
**I put a lot of effort into resisting bad foods**				
Do not agree	1,302 (56.6)	847 (58.8)	455 (53.0)	0.007
Agree	996 (43.3)	592 (41.1)	404 (47.0)	
Did not disclose	1 (<0.1)	1 (<0.1)	0	
**Fat people are unhealthy**				
Do not agree	1,443 (62.8)	888 (61.7)	555 (64.6)	0.185
Agree	843 (36.7)	543 (37.7)	300 (34.9)	
Did not disclose	13 (0.5)	9 (0.6)	4 (0.5)	
**Health Motivation**				
Not motivated	1,116 (48.5)	878 (61.0)	238 (27.7)	<0.001
Motivated	1183 (51.5)	562 (39.0)	621 (72.3)	
**Weight Motivation**				
Not motivated	1,881 (81.8)	1,222 (84.9)	659 (76.7)	<0.001
Motivated	418 (18.2)	218 (15.1)	200 (23.3)	

Table 2 presents the results of the models. Those who agreed that they put a lot of effort into resisting “bad foods” were more likely to limit meat intake (*p* < 0.002). Mediation analyses revealed health-related dietary motivations fully mediated the association, while weight-related dietary motivations partially mediated the association. No relationship was observed between the belief that fat people are unhealthy and meat limitation (*p* = 0.38). However, both health and weight mediated the relationship (*p* < 0.015).

**Table 2:** Relationships between agreement with each diet culture statement and meat limitation, as mediated by health or weight motivations.

Relationship	Estimate	Standard Error	p value
**Moralization -> Health -> Dietary Pattern**			
Path C: Total Effect	0.18	0.06	0.002
Path A (Moralization -> Health)	0.63	0.06	<0.001
Path B (Health -> Dietary Pattern)	0.81	0.06	<0.001
Path AB: Indirect Effect	0.19	0.02	<0.001
Path C': Direct Effect	−0.02	0.06	0.720
**Moralization -> Weight -> Dietary Pattern**			
Path C: Total Effect	0.18	0.06	0.002
Path A (Moralization -> Weight)	0.61	0.06	<0.001
Path B (Weight -> Dietary Pattern)	0.27	0.07	<0.001
Path AB: Indirect Effect	0.04	0.01	0.004
Path C': Direct Effect	0.14	0.06	0.017
**Fat Bias -> Health -> Dietary Pattern**			
Path C: Total Effect	−0.05	0.06	0.380
Path A (Fat Bias -> Health)	0.17	0.06	0.003
Path B (Health -> DP)	0.81	0.06	<0.001
Path AB: Indirect Effect	0.05	0.02	0.006
Path C': Direct Effect	−0.10	0.06	0.066
**Fat Bias -> Weight -> Dietary Pattern**			
Path C: Total Effect	−0.05	0.06	0.380
Path A (Fat Bias -> Weight)	0.21	0.07	0.001
Path B (Weight -> Dietary Pattern)	0.27	0.07	<0.001
Path AB: Indirect Effect	0.02	0.01	0.014
Path C': Direct Effect	−0.07	0.06	0.270

### 3.1 Interviews with meat limiters

Over half of interview participants (61 %, *n* = 17) imparted a moralization of “good” versus “bad” onto foods, with highly processed foods and animal-based foods often referred to using terms such as “unhealthy”, “junk”, or “awful”. Negative views about weight were less frequently mentioned, but still important for participants (25 %, *n* = 7) who equated weight to health.

Just under half of interview participants (*n* = 13) mentioned the healthfulness of plant-based diets. Some (*n* = 8) described health benefits they have experienced while others (*n* = 9) shared the belief that plant-based diets are not inherently healthy, including four people who discussed both. A few (*n* = 3) reported that weight control motivated their decision to limit meat. For example, one participant stated: “I stopped eating meat like over 30 years ago so… I wanted to get my weight under control” (Woman, Pescatarian, 58). Another shared that, while she is not motivated by health or weight, her peers and family associate plant-based eating with health and weight loss: “I think for [my family], even the notion of salads and… plant-based stuff is for people who are fit and healthy… or even just to lose weight” (Woman, Pescatarian, 19).

## 4 Discussion

This study adds to the growing body of research examining the moralization of food and meat limitation [1,[16], [17], [18]], and is relevant to ongoing public and scientific debates about meat consumption, planetary boundaries, climate change, and human health. We find that individuals who seek to resist “bad foods” are more likely to limit meat intake because they perceive it to be better for their health and to promote weight loss. Both our survey and interview data support these findings.

Fear of “bad foods” can negatively impact relationships with bodies and food [1] and may contribute to disordered eating [19]. In fact, restrictive dieting is often a precursor to disordered eating [20]. The moralization of foods and the focus on doing things “right” may come at the expense of overall health [19]. Therefore, clinical and public health discussions around plant-based eating must be approached with nuance as to the benefits of eating a diversity of plant-based foods (e.g., fiber, antioxidants) and acknowledgement for what motivates meat limitation to avoid inadvertently contributing to harmful relationships with food.

We did not find the belief that fat people are unhealthy to be associated with meat limitation. However, mediation was examined since plant-based diets can be used for weight loss or disordered dietary control [21,22]. We found that fat bias is associated with feeling motivated by both health and weight loss which, as noted above, are each associated with limiting meat intake. It is possible that factors that were not considered in our models compete with the motivation variables and reduce the overall relationship such as weight status or social media engagement, both of which have previously been linked to diet culture beliefs [23,24] and food choices [25,26]. It is also possible that participants may have implicit anti-fat bias and are motivated to not become fat themselves even if they do not think fat people are unhealthy.

The mixed methods approach is a marked strength of this study. Limitations relate to the sample and measures. The final survey sample overrepresented older, White women. Future work should incorporate a more diverse group of respondents [11,27]. We used two questions from an unvalidated scale to measure diet culture beliefs [11]. Scale validation is needed to advance research on this important topic.

## 5 Conclusion

As concerns with the interconnected impacts of current food systems and diets on health, the environment, and climate change (sometimes referred to collectively as planetary health) increase, the focus on reducing meat consumption has grown. This study examined the relationship between diet culture beliefs and meat limitation. The results suggest that health and weight loss goals can act as a bridge between the moralization of food and meat limitation. Thus, reducing meat consumption could be a way of enacting unhealthy relationships with food. These findings emphasize the need for clinicians and public health professionals to avoid moralizing foods and focusing on body size and shape when discussing the attributes of plant-based eating patterns.

## CRediT authorship contribution statement

**Marina F. Jiao:** Writing – original draft, Investigation, Conceptualization. **Saadatu Abdul-Rahaman:** Writing – review & editing, Supervision. **Michelle Leonetti:** Writing – review & editing, Supervision, Investigation. **Lizzy Pope:** Writing – review & editing, Conceptualization. **Kelsey Rose:** Writing – review & editing. **Emily H. Belarmino:** Writing – review & editing, Supervision, Methodology, Funding acquisition, Conceptualization.

## Declaration of competing interest

The authors declare that they have no known competing financial interests or personal relationships that could have appeared to influence the work reported in this paper.

## Appendix Supplementary materials

Image, application 1

Image, application 2

## Footnotes

Supplementary material associated with this article can be found in the online version at doi:10.1016/j.joclim.2025.100461.
