# Supplementary material for: Diet culture mindset and meat restriction: A mixed methods mediation analysis
Source: J Clim Chang Health. 2025 Jun 10;23:100461. doi: 10.1016/j.joclim.2025.100461 (PMC12851305; doi:10.1016/j.joclim.2025.100461)
Supplement: Supplementary file 1 [file mmc1.docx]

**SUPPLEMENTAL TABLES**

Table 1: Survey Questions and Associated Variables

| Survey Question | Response Options | Recoded Variables |
| --- | --- | --- |
| Which of the following dietary patterns do you most closely identify with? | - Omnivore (do not limit red meat, poultry, and/or fish) - Flexitarian (intentionally limit red meat, poultry, and/or fish) - Pescatarian (eat fish, eggs and/or dairy, but not red meat or poultry) - Vegetarian (do not eat red meat, poultry, or fish, but consume eggs and/or dairy) - Vegan (do not eat any animal products) | Binary:   - 0 - Omnivore (omnivore) - 1- Plant-based (Flexitarian, pescatarian, vegetarian, and vegan) |
| What are your reasons for following this dietary pattern? Select all that apply. | - Environment - Ethics/Animal welfare - Health - I was raised this way - My friends and family eat this way - Religious/Spiritual belief - Taste - To save money - Weight management - Other _________ | Only health and weight used  Health   - 0 – Not health motivated - 1 – Health motivated   Weight   - 0 – Not weight motivated - 1 – Weight motivated |
| Please indicate your level of agreement with each statement on a scale of 1–5 with 1 being strongly disagree and 5 being strongly agree.  “Fat people are unhealthy” | 1. Strongly disagree 2. Disagree 3. Neither agree nor disagree 4. Agree 5. Strongly agree | - 0 – Do not agree (1, 2, 3) - 1 – Agree (4, 5) |
| Please indicate your level of agreement with each statement on a scale of 1–5 with 1 being strongly disagree and 5 being strongly agree.  “I put a lot of effort into resisting bad foods” | 1. Strongly disagree 2. Disagree 3. Neither agree nor disagree 4. Agree 5. Strongly agree | - 0 – Do not agree (1, 2, 3) - 1 – Agree (4, 5) |
| Please select your gender | - Woman - Man - Non-binary/non-conforming - Prefer not to answer | - 0 – Not a woman (man, non-binary, no answer) - 1 – Woman (woman) |
| Which of the following best describes your household income range in 2022 before taxes?    In this survey, a ‘household’ is a group of people who share a private dwelling and normally spend four or more nights a week in the household. Household members must share consumption of food or contribute some portion of income towards the costs for living as a group. | - Less than $24,999 per year - $25,000-$74,999 per year - $75,000-$99,999 per year - $100,000 to $149,999 - $150,000 to $199,999 - $200,000 or more | - 0 – Less than 75k (less than $24,999; $25,000-$74,999) - 1 – 75k or more ($75,000-$99,999; $100,000-$149,999; $150,000-$199,999; $200,000 or more) |
| What is your race? Select all that apply | - American Indian or Alaskan Native - Black or African American - Asian - Native Hawaiian or Other Pacific Islander - White - Other race or origin | - 0 – Not BIPOC (White) - 1 – BIPOC (American Indian/Alaskan Native; Black or African American; Asian; Native Hawaiian; Hispanic, Latino, or Spanish) |
| What is the highest level of formal education that you have completed? | - Some high school (no diploma) - High school graduate (including GED) - Some college (no degree) - Associates degree/technical school/apprenticeship - Bachelor’s degree - Postgraduate (e.g. Masters, PhD) / professional degree (e.g. JD) | - 0 – Up to an associate’s (some high school, high school grad, some college, associates degree) - 1 – Bachelor’s or more (Bachelor’s, postgraduate) |

Table 2: Overall assessment of mediation using analysis of deviance

| Relationship | Variables | Chi-Square | p-value |
| --- | --- | --- | --- |
| Moralization -> Health |  |  |  |
| Unmediated | **Moralization** | **10.1** | **0.002** |
|  | **Education** | **32.3** | **<.001** |
|  | **Gender** | **10.9** | **<.001** |
|  | **Age** | **36.8** | **<.001** |
|  |  |  |  |
| Path A | **Moralization** | **128.3** | **<.001** |
|  | **Education** | **20.4** | **<.001** |
|  | Gender | 3.07 | 0.08 |
|  | **Age** | **26.7** | **<.001** |
|  |  |  |  |
| Path B | **Health motivation** | **199.7** | **<.001** |
|  | **Education** | **20.5** | **<.001** |
|  | **Gender** | **8.48** | **0.004** |
|  | **Age** | **24.8** | **<.001** |
|  |  |  |  |
| Mediation | Direct effect | 0.13 | 0.717 |
|  | **Indirect effect** | **82.8** | **<.001** |
|  | **Education** | **31.1** | **<.001** |
|  | **Gender** | **9.13** | **0.003** |
|  | **Age** | **30.6** | **<.001** |
| Moralization-> Weight |  |  |  |
| Unmediated | **Moralization** | **10.1** | **0.002** |
|  | **Education** | **32.3** | **<.001** |
|  | **Gender** | **10.9** | **<.001** |
|  | **Age** | **36.8** | **<.001** |
|  |  |  |  |
| Path A | **Moralization** | **90.6** | **<.001** |
|  | Education | 1.44 | 0.231 |
|  | Gender | 1.38 | 0.24 |
|  | **Age** | **11.1** | **<.001** |
|  |  |  |  |
| Path B | **Weight motivation** | **14.8** | **<.001** |
|  | **Education** | **31.6** | **<.001** |
|  | **Gender** | **10.6** | **0.001** |
|  | **Age** | **31.4** | **<.001** |
|  |  |  |  |
| Mediation | **Direct effect** | **5.65** | **0.017** |
|  | **Indirect effect** | **8.31** | **0.004** |
|  | **Education** | **28.3** | **<.001** |
|  | **Gender** | **10.4** | **0.001** |
|  | **Age** | **30.7** | **<.001** |
| Fat Bias -> Weight |  |  |  |
| Unmediated | Fat bias | 0.77 | 0.38 |
|  | **Education** | **32.8** | **<.001** |
|  | **Gender** | **10** | **0.002** |
|  | **Age** | **33.9** | **<.001** |
|  |  |  |  |
| Path A | **Fat bias** | **10.2** | **0.001** |
|  | Education | 1.08 | 0.3 |
|  | Gender | 3.8 | 0.05 |
|  | **Age** | **5.46** | **0.02** |
|  |  |  |  |
| Path B | **Weight motivation** | **14.8** | **<.001** |
|  | **Education** | **31.5** | **<.001** |
|  | **Gender** | **10.6** | **0.001** |
|  | **Age** | **32** | **<.001** |
|  |  |  |  |
| Mediation | Direct effect | 1.23 | 0.27 |
|  | **Indirect effect** | **6.08** | **0.01** |
|  | **Education** | **30.4** | **<.001** |
|  | **Gender** | **9.78** | **0.002** |
|  | **Age** | **32.4** | **<.001** |
| Fat Bias -> Health |  |  |  |
| Unmediated | Fat bias | 0.77 | 0.38 |
|  | **Education** | **32.8** | **<.001** |
|  | **Gender** | **10** | **0.002** |
|  | **Age** | **33.9** | **<.001** |
|  |  |  |  |
| Path A | **Fat bias** | **8.64** | **0.003** |
|  | **Education** | **20.7** | **<.001** |
|  | **Gender** | **5.99** | **0.014** |
|  | **Age** | **16.9** | **<.001** |
|  |  |  |  |
| Path B | **Health motivation** | **195.8** | **<.001** |
|  | **Education** | **20.3** | **<.001** |
|  | **Gender** | **8.56** | **0.003** |
|  | **Age** | **25.1** | **<.001** |
|  |  |  |  |
| Mediation | Direct effect | 3.38 | 0.066 |
|  | **Indirect effect** | **7.5** | **0.006** |
|  | **Education** | **35.1** | **<.001** |
|  | **Gender** | **9.88** | **0.002** |
|  | **Age** | **33.4** | **<.001** |

Bold indicates inclusion in the final models.

Table 3: Demographic variables and their significance within diet culture and diet pattern model

|  | Moralization | Fat Bias |
| --- | --- | --- |
| Racial Identity | 0.793 | 0.901 |
| **Age** | **<.001** | **<.001** |
| **Gender** | **<.001** | **<.001** |
| **Education** | **<.001** | **<.001** |
| Income | 0.038 | 0.029 |

Bold indicates inclusion in the final models.
