# Supplementary material for: Diet culture mindset and meat restriction: A mixed methods mediation analysis
Source: J Clim Chang Health. 2025 Jun 10;23:100461. doi: 10.1016/j.joclim.2025.100461 (PMC12851305; doi:10.1016/j.joclim.2025.100461)
Supplement: Supplementary file 2 [file mmc2.docx]

Understanding factors associated with plant-based diets among rural Americans – phase 2

**GUIDE FOR SEMI-STRUCTURED INTERVIEWS**

**Note, this guide presents interview topics as well as draft questions. Questions may be modified based on the nature of the interview and participant responses.

*Thank you for completing the survey. In this interview, I am going to ask you some questions about foods that you eat and enjoy and your thoughts on eating a plant-based diet in a rural community.*

**Interview Questions:**

- **Opening Questions that Develop Rapport**:
  - Can you tell me about what you ate for dinner last night and whether this is a “typical” meal for you?
    - Probe: If not, what would be considered typical?
  - Overall, how would you describe the way that you eat?
- **SHOW DIAGRAM OF A PLANT-BASED DIET (Appendix A)**
  - During this interview we are going to use the term ‘plant-based diet’. When we use the term diet, we are not referring to calorie restriction related to weight loss. But we are talking about a pattern of eating. **We define a plant-based diet as** a dietary pattern dominated by fresh or minimally processed plant foods and decreased consumption of meat, eggs and dairy products. Compared to meat-centered diets, plant-based diets involve increased consumption of a variety of grains, fruits, vegetables, beans, nuts and seeds. So it does not necessarily mean a vegetarian diet and can contain modest amounts of fish, meat, and dairy. (Wyker and Davison 2010)
  - Based on the way that you answered questions on our survey, we have categorized the way you eat as ‘plant-based’
- **Personal Perspectives and Experiences of Eating Plant-Based**:
  - I’m curious to learn more about why you eat a plant-based dietary pattern. What are some of the reasons that you do it?
  - What was your experience starting a plant-based dietary pattern?
    - Probe: How long have you been eating this way?
  - On a 10-point scale from ‘very easy’ to ‘very difficult’, how would you categorize your personal experience eating a plant-based diet in rural <VERMONT/TEXAS>?
    - Probe: What made you choose this number?
    - Probe: What factors make it easy?
    - Probe: What factors make it difficult? (cost, time, availability, seasonality, etc?)
- **Outside Perceptions of Plant-Based Diet:**
  - What do your friends and family think about the way that you eat?
    - Probe: Do you ever get pushback?
    - Probe: Do the people around you eat in similar ways?
  - What do you think other people in your community think about plant-based diets?
- **Community Recommendations**:
  - If you were to give tips to a friend or a neighbor about how to eat a plant-based diet, what would you say?
    - Probe: Do you have tips, hacks or recommendations that make eating a plant-based diet easier in general?
  - If you could make a change in your community that would make it easier for other people to eat plant-based, what would it be?
    - Probe: Tell me more about that.
    - Probe: How would you suggest that change gets implemented?
- **Questions about nutrients of concern**:
  - Do you have any concerns about the healthfulness of a plant-based diet?
    - Probe: Tell me about them.
    - Probe: Are there any nutrients that you are concerned about on a plant-based diet?
  - What do you think about when choosing plant-based protein foods?
    - Probe: What are your thoughts on plant-based meat alternatives?
  - Do you seek out any specific foods or food combinations to fill in possible gaps in your diet from avoiding/eating very little animal products?
    - Probe: I am interested to hear more about that.
  - ***Specific questions based on responses to the survey such as:***
    - I see that you take Vitamin B12; can you talk about your decision to take this supplement?
    - I see that you eat nutritional yeast more than once a week; can you talk about the reasons why?
    - Etc…
- **Closing questions**:
  - Is there anything else about eating a plant-based diet in your community that you would like to share?
  - Is there anything else about diet planning that we haven’t talked about that you would like to mention?

Appendix 1. Plant-based diet plate


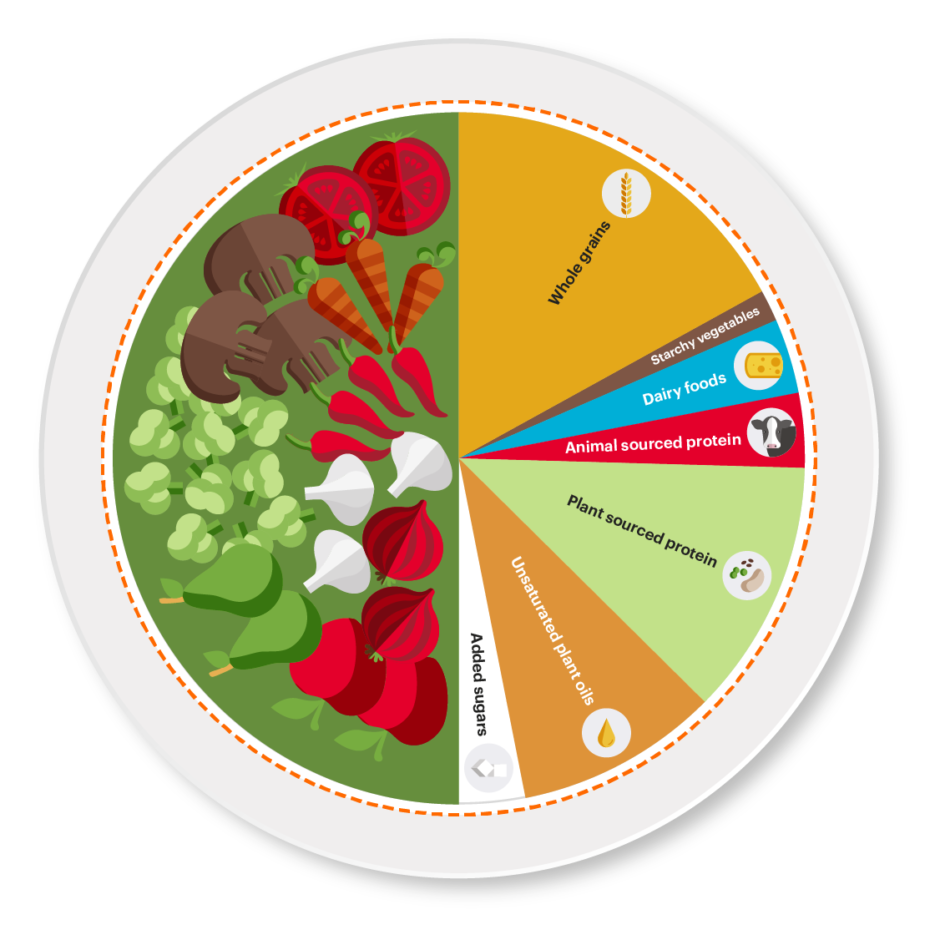


© EAT-Lancet Commission on Food, Planet, Health

https://eatforum.org/eat-lancet-commission/the-planetary-health-diet-and-you/
